# Ingested Fish Bone Lodged in the Vallecula

**DOI:** 10.7759/cureus.8761

**Published:** 2020-06-22

**Authors:** Mitchell McElroy, Latha Ganti, Jessica Houck, Amanda L Webb, David Lebowitz

**Affiliations:** 1 Emergency Medicine, University of Central Florida College of Medicine, Orlando, USA; 2 Emergency Medicine, Envision Physician Services, Nashville, USA; 3 Emergency Medicine, University of Central Florida College of Medicine/Hospital Corporation of America Graduate Medical Education Consortium of Greater Orlando, Orlando, USA; 4 Emergency Medical Services, Polk County Fire Rescue, Bartow, USA; 5 Emergency Medicine, University of Central Florida College of Medicine/Hospital Corporation of America Graduate Medical Education Consortium, Kissimmee, USA

**Keywords:** vallecula, fish bone

## Abstract

Although foreign body ingestions are less common in adults than children, when they do occur, it is often due to a fish or chicken bone. The authors present a case of a fish bone ingestion, and highlight its appearance on imaging.

## Introduction

Pain from an ingested foreign body is a common emergency department (ED) chief complaint. Of adult patients complaining of foreign body ingestion, only a fraction demonstrate clear foreign body impaction, with the remainder finding spontaneous symptom relief in 48 hours [1]. Although foreign body impaction is much more common in young children than adults, fish bones are among the most common offending foreign bodies in adults [2-4]. Ingested fish bones may go undiagnosed due to paucity of symptoms or diagnostic uncertainty and may lead to severe complications, such as gastrointestinal perforation or retropharyngeal abscess [5]. This case reports an emergency presentation of a fish bone ingestion along with discussion of radiologic findings and diagnosis.

## Case presentation

This is a 29-year-old female who presented to the ED with the chief complaint that she had something stuck in her throat. She was out with her husband having dinner and felt that a fish bone was stuck in her throat. This happened 30 minutes prior to the ED arrival. She complained of neck pain. The pain was worsened by speaking or swallowing. She was able to speak in full sentences. She denied feeling short of breath. The patient’s vital signs upon arrival were temperature 98.9^0^F, pulse 73 beats per minute, blood pressure 134/91 mmHg, and oxygen saturation 100%. She denied any medical or surgical history, had no allergies to medications, and denied smoking or illicit drugs. She endorsed drinking alcohol on occasion. On physical examination, she was a well-developed well-nourished female in no acute distress except for mild anxiety due to the foreign body sensation. Her airway was patent, she had moist mucous membranes, and intact dentition. She was salivating, almost in an effort to dislodge the foreign body on her own. Heart and lung sounds were normal, and the rest of her physical examination was unremarkable.

**Figure 1:**
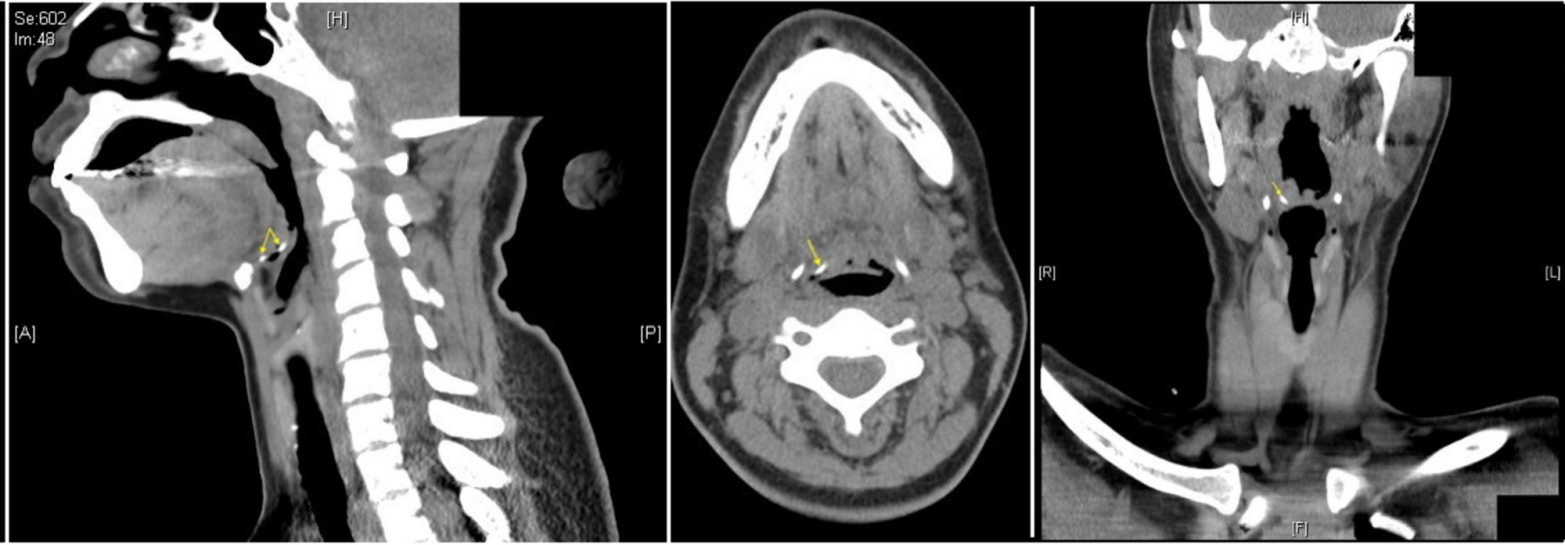
CT panel demonstrating the fish bone right vallecula (arrows).

A CT scan of the neck was obtained (Figure 1) which demonstrated a curvilinear 2-cm radiopaque foreign body in the right vallecula.

The patient was admitted to the hospital for ear, nose, throat (ENT) consultation and removal of the foreign body. It was indeed a fish bone and was successfully removed the following morning without any complications.

## Discussion

Diagnosis of an ingested fish bone may be difficult to make due to several factors. Patients may present in great variations of time from onset; some patients may present weeks to even months after onset of symptoms [2,6,7]. This variation in the time to presentation may reduce clinician suspicion of foreign body. This is especially the case due to the variable rate of true foreign bodies found after patients presenting with sharp pain in the throat following ingestion of fish. One study found that as many as 79% of patients presenting with this chief complaint had no foreign body found during workup [1]. Although rare, lethal complications of ingested foreign bodies mandate that clinicians maintain a high index of suspicion. Undiagnosed ingested fish bones may lead to perforation of the gastrointestinal tract leading to compromise of the local vasculature, including the carotids and the aorta, retropharyngeal or other cervical abscess, pneumonia, mediastinitis, or pneumothorax [3,5,7].

A complete history and physical exam are the first high-value data for the clinician to consider. Findings such as odynophagia, dysphagia, and drooling are relatively sensitive for an ingested fish bone [6,8]. History of fish consumption is an obvious contributor; however, it is important to consider differing cultural practices in fish consumption that may elevate the risk of fish bone ingestion, as some people customarily cook and consume fish with bones intact [2]. If clinical suspicion remains high, fiberoptic laryngoscopy is the preferred next step in diagnosis, but a lateral neck radiograph is normally obtained in addition or substitute [9]. Any obvious foreign body, inappropriate gas or fluid in soft tissue, or prevertebral soft-tissue swelling should raise suspicion for an ingested foreign body [9]. Lateral neck radiographs have two major disadvantages. First, fish bones have a wide spectrum of radiodensity; therefore, even a negative radiograph has a low negative predictive value because of the possibility of a radiolucent fish bone [8]. In fact, one study reported that 21 of 28 fish or meat bones remained undiagnosed by radiograph [6]. Second, normal calcification or ossification of the styloid process, styloid ligament, tracheal cartilage, thyroid cartilage anterior longitudinal ligament, spinal osteophytes, sialolith, vasculature, or lymph tissue may lead to a false-positive radiograph and potentially unnecessary procedures [8,9].

Because of these scenarios, CT and flexible laryngoesophagoscopy are the superior diagnostic modalities for definitive diagnosis [9,10]. In the case that CT identifies a foreign body, a novel method for risk stratification of these patients can be made by shape of the foreign body. Although all foreign bodies should be removed, pin and spindle-shaped foreign bodies have higher rate of perforation and severe complications when compared to other shapes with more points of contact [3]. If CT does not identify a foreign body or other suspicious abnormality, patients are often managed conservatively with education to return to the hospital in the event of fever or worsening of symptoms, as mucosal abrasion may lead to the same clinical presentation and usually resolves in 48 hours [1,9].

## Conclusions

This case demonstrates the potential difficulty in the diagnosis of ingested fish bones, and the benefit of expedient diagnosis in the ED. Although rare, severe complications may arise in the case of delayed diagnosis or treatment. The milieu of nonspecific history and physical findings in conjunction with radiologic challenges requires the clinician to maintain a high index of suspicion and a reasonably low threshold for ENT consultation in the event of negative radiologic testing with a suspicious clinical presentation.

## References

[1] Knight LC, Lesser TH (1989). Fish bones in the throat. Arch Emerg Med.

[2] Kim SY, Park B, Kong IG, Choi HG (2016). Analysis of ingested foreign bodies according to age, type and location: a retrospective observational study. Clin Otolaryngol.

[3] Ruan W-S, Li Y-N, Feng M-X, Lu Y-Q (2020). Retrospective observational analysis of esophageal foreign bodies: a novel characterization based on shape. Sci Rep.

[4] Li Z-S, Sun Z-X, Zou D-W, Xu G-M, Wu R-P, Liao Z (2006). Endoscopic management of foreign bodies in the upper-GI tract: experience with 1088 cases in China. Gastrointest Endosc.

[5] Swain SK, Singh N, Sahu MC (2016). An unusual presentation of fish bone ingestion in an adolescent girl: a case report. Egypt J Ear Nose Throat Allied Sci.

[6] Orji FT, Akpeh JO, Okolugbo NE (2012). Management of esophageal foreign bodies: experience in a developing country. World J surg.

[7] Wu E, Huang L, Zhou Y, Zhu X (2018 ). Migratory fish bone in the thyroid gland: case report and literature review. Case Rep Med.

[8] Ng SJK, Lee JKT, Thian YL (2018). Cricoid ridge ossification mimicking ingested fish bone on plain radiography: prevalence in Singapore. Singapore Med J.

[9] Castán Senar A, Dinu LE, Artigas JM, Larrosa R, Navarro Y, Angulo E (2017). Foreign bodies on lateral neck radiographs in adults: imaging findings and common pitfalls. RadioGraphics.

[10] Palme CE, Lowinger D, Petersen AJ (1999). Fish bones at the cricopharyngeus: a comparison of plain-film radiology and computed tomography. Laryngoscope.

